# Costal cartilage fracture: A commonly missed thoracic injury in trauma patients

**DOI:** 10.1016/j.radcr.2021.10.008

**Published:** 2021-11-01

**Authors:** Moussa Meteb, Bader Abou Shaar, Ghassan Awad El-Karim, Youssef Almalki

**Affiliations:** a The Ottawa Hospital Research Institute, 501 Smyth Rd, Ottawa, ON, K1H 8L6, Canada; b Alfaisal University, College of Medicine, Riyadh, Saudi Arabia; c University of Toronto, Faculty of Medicine, Toronto, ON, Canada; d Department of Diagnostic Imaging, Bluewater Health, Sarnia ON, Canada

**Keywords:** Costal Cartilage Fracture, Costochondral Injury, Blunt Chest Trauma, Rib Injury, Emergency Radiology

## Abstract

In this report, we describe a case of a 43-year-old male who sustained a displaced mid chondral costal cartilage fracture along with multiple ipsilateral rib fractures following a jet ski collision. Costal cartilage fractures (CC) are commonly missed on imaging evaluation, and accordingly underreported in the literature. High-energy blunt chest trauma represents the most common mechanism for CC injuries. Computed Tomography (CT) is the modality of choice to diagnose CC fractures along with the associated cardiopulmonary and abdominal injuries in polytrauma patients. There is currently no consensus with respect to the management of CC fractures. Further research is required to explore the long-term impact of CC fractures on thoracic cage stability.

## Introduction

Thoracic injuries are a significant cause of morbidity and mortality in trauma patients, accounting for nearly 35% of trauma-related deaths in the United States [1]. Motor vehicle collisions (MVCs) are the most common cause of blunt chest trauma [2,3], with chest injuries including single or multiple rib fractures, lung contusion, pneumothorax, hemothorax, flail chest, heart or vessel injury, sternal fractures, and/or injuries to the airways and thoracic spine [4].

Costal cartilage (CC) fractures are commonly missed on imaging evaluation, and accordingly underreported in the literature [5]. The incidence of CC fractures is estimated to be between 20%-42%, primarily secondary to high-energy blunt chest trauma (eg, MVCs, falls, sports injuries), and often co-existent with multiple consecutive rib fractures [5,6]. Recognition of CC fractures is important to ensure stability of the thoracic cage.

In this report, we describe a case of a 43-year-old male with costal cartilage and rib fractures following high-energy blunt chest trauma. A brief description of the current literature on costal cartilage injuries is also provided.

## Case

**Fig. 1:**
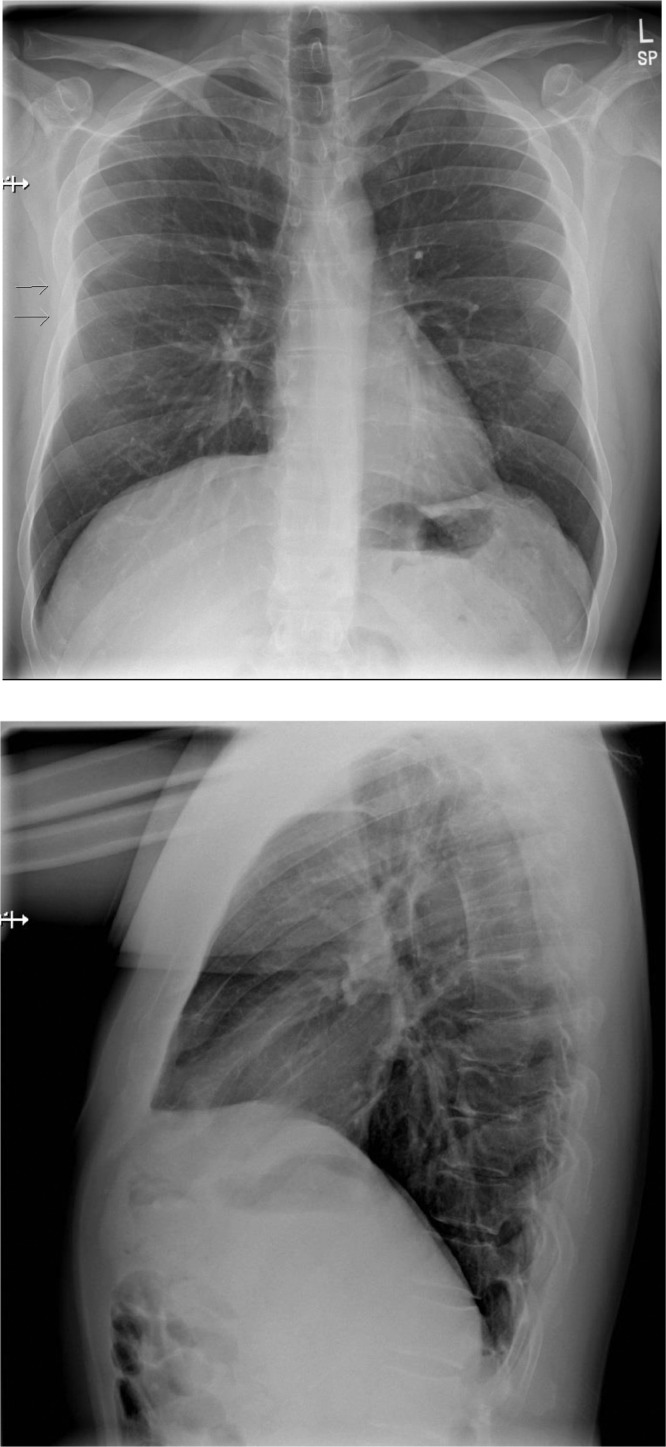
PA and lateral chest radiographs. PA chest radiograph demonstrates subtle asymmetric deformity of the right rib cage starting from the 6th rib (arrow).

**Fig. 2:**
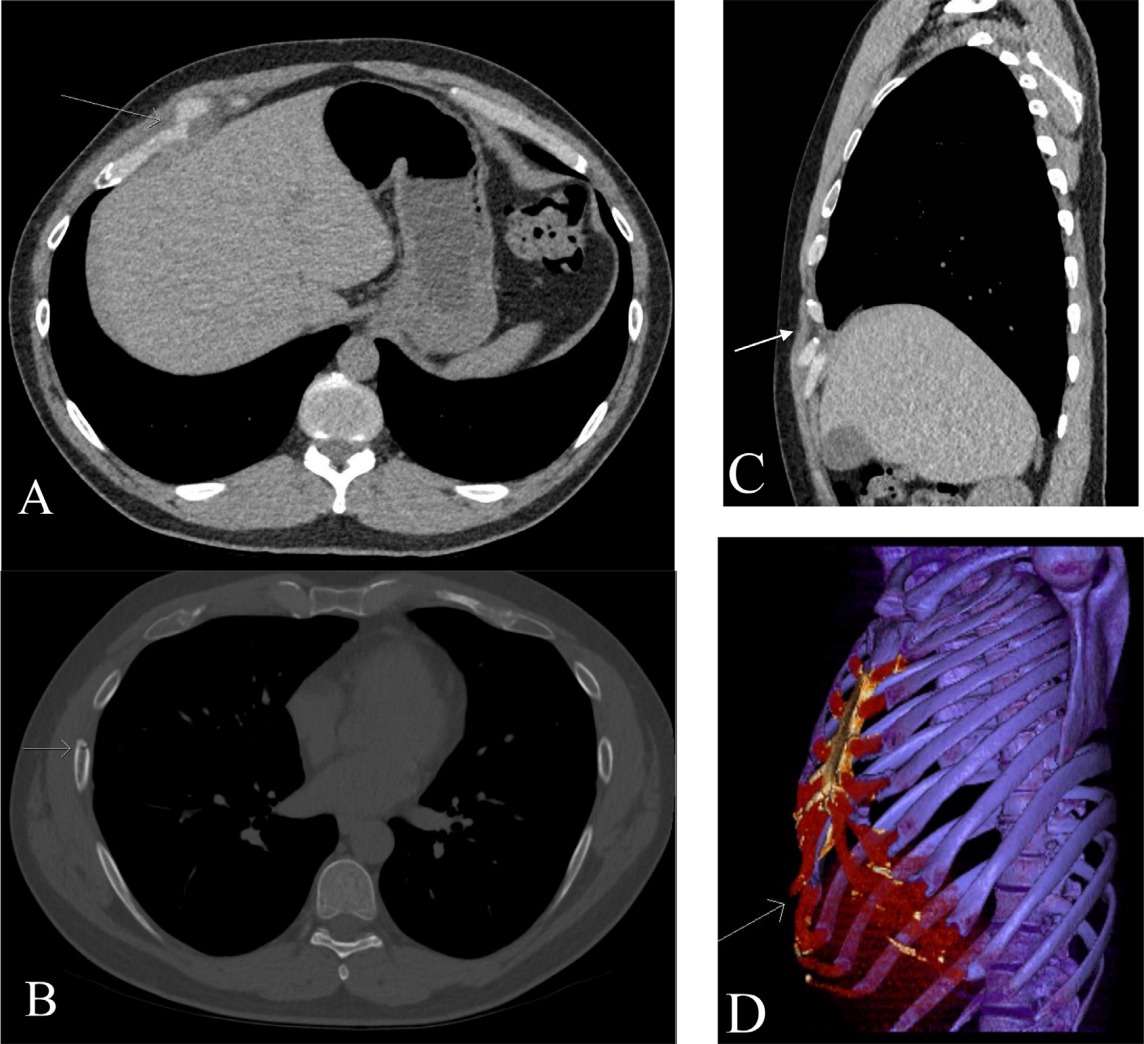
Low-dose CT chest with multiple reformats and 3D reconstruction. (A) Soft-tissue window demonstrates a displaced 7th mid chondral costal cartilage fracture (arrow). (B) Bone window demonstrates ipsilateral nondisplaced anterior 5th rib fracture and minimally distracted lateral 7th rib fracture (arrows). (C) Sagittal soft-tissue window of displaced 7th mid chondral costal cartilage fracture and (D) associated 3D reconstruction.

A 43-year-old male hospital executive presented to his physician complaining of right anterior chest pain three days following a Jet Ski incident where he was a passenger ejected while the driver performed a 90 degree sharp turn. The patient continued in the initial forward direction while the Jet Ski turned. The patient landed onto the water laterally onto his right hemithorax with his arm abducted above his head. He described difficulty breathing initially with the lower anterolateral hemithorax feeling deformed in a concave manner. By the time he was taken to shore, the concave deformity had resolved, and he was able to breathe with less difficulty. He presented to his physician due to his need for continuous analgesia use and trouble sleeping due to pain. He also described thoracic pain with right arm abduction and with inspiration. Initial physical examination revealed focal tenderness at the right anterior 6th and 7th rib region. A chest radiograph was requested with reason for examination being “trauma to the chest with pleuritic chest pain, rule out rib fracture and lung injury” (Fig. 1). The cardio mediastinal contour was normal. The lungs were well aerated. There was no pneumothorax or pleural effusion. No mediastinal shift. No displaced rib fractures were identified. A slight deformity was noted on the Chest X-ray centered around the 6th and 7th rib with the right hemithorax slightly medial displaced. Low-dose CT chest was performed for further evaluation (Fig. 2), which revealed a mildly displaced 7th mid chondral costal cartilage fracture, along with a non-displaced anterior 5th rib fracture, and a minimally distracted anterolateral 7th rib fracture. The patient was prescribed analgesics for pain control with an outpatient referral to Orthopaedic Surgery. The outcome of the referral was to manage the patient conservatively with follow-up to reassess healing and/or union and need for future surgical management.

## Discussion

CC fractures are frequently missed on initial CT imaging evaluation with a low detection rate of 39.5% in a retrospective analysis of 1461 trauma patients in a level 1 trauma center [5]. High-energy blunt chest trauma represents the most common mechanism for CC injuries, with an incidence between 20%-42% [5,6]. CC fractures can be categorized as either mid chondral, costochondral, or chondrosternal depending on the site of injury, with most fractures involving the costal cartilages of the seventh (21%), first (17%), and sixth rib (15%) [5]. Nummela et al. (2017) showed that multiple consecutive rib fractures, sternal fractures, pneumothorax, hemothorax, aortic injury, thoracic spine fractures and hepatic injury were more commonly seen in patients with CC injuries than in patients without CC fractures. Interestingly, there was no significant difference in the mechanisms of injury between the two chest trauma groups to account for these observations.

Unlike osseous rib fractures, CC fractures cannot be detected with conventional chest radiography unless there is a strong calcification of the involved cartilage which can be seen in cases of irregular ossification foci that appear in adulthood. CT, MRI and even ultrasound have been described as reliable imaging modalities to diagnose CC injuries [7,8]. Multidetector computed tomography, which is the workhorse for imaging evaluation of polytrauma patients, can also identify the concomitant injuries described above which commonly occur with CC fractures following high-energy blunt chest trauma. In our case, the mid chondral 7th rib costal cartilage fracture and associated anterior 5th and lateral 7th rib fractures were inconspicuous on chest radiograph with only a slight local asymmetric deformity. A definitive diagnosis of costal cartilage injury with associated multiple right sided rib fractures was made on low-dose CT to explain the patient's symptoms.

There is currently no consensus with respect to the management of CC injuries, particularly since they are underreported in literature. The majority of patients are managed conservatively with analgesia [5]. In some cases, patients may undergo surgical plate fixation [9,10]. The natural prognosis of CC fractures is also unknown, particularly whether these fractures will heal to form a stable union or remain a concern for possible instability of the thoracic wall [11]. Patients with CC injuries and multiple consecutive rib fractures may also require ventilator support akin to the management of flail chest. Therefore, increasing familiarly with CC fractures is important for the complete assessment of polytrauma patients, understanding the long-term sequelae of CC injuries on thoracic cage stability and cardiopulmonary function, as well as establishing consensus guidelines for the management of CC fractures.
